# Ultrasound-guided steroid tendon sheath injections in juvenile idiopathic arthritis: a 10-year single-center retrospective study

**DOI:** 10.1186/s12969-017-0155-3

**Published:** 2017-04-11

**Authors:** Shannon E. Peters, Ronald M. Laxer, Bairbre L. Connolly, Dimitri A. Parra

**Affiliations:** 1 School of Medicine and Medical Sciences, University College Dublin, Dublin, Ireland; 2 Division of Rheumatology, Department of Pediatrics and Medicine, The Hospital for Sick Children and University of Toronto, Toronto, ON Canada; 3 Image Guided Therapy, Department of Diagnostic Imaging, The Hospital for Sick Children and University of Toronto, Toronto, ON Canada

**Keywords:** Tenosynovitis in children, Steroid injections, Tendon sheath, US-guidance, JIA

## Abstract

**Background:**

The aims of this study were to: (a) Identify tendon sheaths most commonly treated with steroid injections in a pediatric patient population with Juvenile Idiopathic Arthritis (JIA); (b) Describe technical aspects of the procedure; (c) Characterize sonographic appearance of tenosynovitis in JIA; (d) Assess agreement between clinical request and sites injected.

**Methods:**

This was a 10 year single-center retrospective study (May 2006-April 2016) of patients with JIA referred by Rheumatology for ultrasound-guided tendon sheath injections. Patient demographics, clinical referral information, sonographic appearance of the tendon sheaths and technical aspects of the procedure were analyzed.

**Results:**

There were 308 procedures of 244 patients (75% female, mean age 9.6 years) who underwent a total of 926 tendon sheath injections. Ankle tendons were most commonly injected (84.9%), specifically the tendon sheaths of tibialis posterior (22.3%), peroneus longus (20%) and brevis (19.7%). The majority of treated sites (91.9%) showed peritendinous fluid and sheath thickening on ultrasound. There were 2 minor intra-procedure complications without sequelae. A good agreement between clinical request and sites injected was observed.

**Conclusions:**

Ultrasound-guided tendon sheath injections with steroids are used frequently to treat patients with JIA. It is a safe intervention with a high technical success rate. The ankle region, specifically the medial compartment, is the site most commonly injected in this group of patients. The most common sonographic finding is peritendinous fluid and sheath thickening. These findings might assist clinicians and radiologists to characterize and more effectively manage tenosynovitis in patients with JIA.

## Background

Juvenile Idiopathic Arthritis (JIA) is the most common chronic rheumatic disease in childhood with an incidence ranging from 1 to 22 per 100,000 [1]. JIA is defined as persistent arthritis for more than 6 weeks with an onset at less than 16 years of age, after excluding other causes of joint inflammation [2]. The etiology of JIA appears to be multi-factorial, and may be related to genetic factors associated with triggering events such as psychological stress, abnormal hormone levels, trauma or infections [1]. JIA includes seven subtypes of arthritis, according to the clinical features during the first 6 months of disease, and with the following frequencies: oligoarticular JIA (50–60%), polyarticular JIA—both rheumatoid factor positive and negative—(30–35%), systemic JIA (10–20%), juvenile psoriatic arthritis (2–15%), enthesitis-related arthritis (1–7%) and undifferentiated arthritis [1]. The knee is the joint most commonly involved in JIA (77%), followed by the ankle (58%) [3], however, many other joints can be affected and become symptomatic including those in the hands, wrists, feet as well as the hips and temporomandibular joints.

Tenosynovitis is one of the manifestations of JIA, and its persistence after adjacent joint injections can explain a lack of clinical response to intra-articular injections. The histopathology of tenosynovitis is indistinguishable from joint synovitis in rheumatoid arthritis (RA). Both are characterized by synovial cell hyperplasia, infiltration by inflammatory cells including lymphocytes and plasma cells, and increased vascularity [4]. Tenosynovitis has been found in up to 71% of JIA patients with symptomatic ankle inflammation [5]. A recent study using MRI found that the most common ankle tendon involved in patients with JIA was the tibialis posterior and an average of 3.5 tendons were involved when tenosynovitis was present [6].

Clinically, tenosynovitis can be suspected by swelling, pain or tenderness along the length of the tendon. On ultrasound (US), an increase of fluid within the synovial sheath, hyperemia and sheath thickening are the most characteristic features of tenosynovitis [7]. The OMERACT Ultrasound Task Force defined tenosynovitis in RA and other inflammatory arthritis as hypoechoic or anechoic thickened tissue with or without fluid in the tendon sheath [8]. This definition is utilized in the RA literature; however to the best of our knowledge, there is little literature reporting the sonographic findings of tenosynovitis in JIA.

Tendon sheath steroid injections are one of the treatment options for tenosynovitis and can be performed in conjunction with intra-articular injections. Utilizing US guidance for steroid injections into tendon sheaths has shown clinical advantage to conventional blind injections in the adult RA population [9], and this may be true in the pediatric population.

In our practice, a large population of patients with JIA is referred to interventional radiology (IR) for joint and tendon sheath injections. However, not uncommonly tendon sheath abnormalities are not seen on US at the time of the procedure. This is consistent with other studies that report discordance between clinical examination of the tendons and imaging findings of tenosynovitis [10, 11]. One study reported tenosynovitis on MRI in more than half of their patient population, whereas no tendon involvement had been detected clinically [6]. This is significant as without the presence of peritendinous fluid, safe tendon sheath injections are difficult to perform.

To the best of our knowledge there is limited literature reviewing the incidence, appearance and management of tenosynovitis in children with JIA. This prompted us to review our experience in the management of patients with JIA and tenosynovitis, referred for tendon sheath steroid injections. The aims of the study were to identify the tendon sheaths most commonly injected in our patient population with JIA, describe the technical aspects of the procedure, characterize the sonographic appearance of tenosynovitis in JIA, and assess the agreement between the clinical request and sites injected.

## Methods

Institutional Research Ethics Board approval was obtained for this study. A 10-year single center retrospective review was completed for all cases of US-guided tendon sheath injections between May 2006 and April 2016. Patients were identified through the Picture Archiving and Communication System (PACS) (General Electric Centricity, GE Healthcare Canada, Mississauga, Ontario). Patients were included if they had a confirmed diagnosis of JIA and if one or more tendon sheath injections were requested by a rheumatologist, with or without joint injections. Patients were excluded if referral or procedural information was incomplete.

Patient demographics and referral information were collected which included the tendon sheaths and steroid dose requested. Each procedural visit to the IR suite was identified as a separate patient encounter, irrespective of the number of tendons and joints injected, and was counted as a “procedure”. The number of prior procedures was also recorded. The procedural data included the type of sedation, the joints and/or tendon sheaths injected and the respective dose of steroid. Each tendon sheath injected was counted individually (i.e. bilateral tibialis posterior injections counted as two injections). If a requested tendon sheath was not injected, the reason was recorded. The number of joints injected was also recorded for each procedure. The procedural imaging and radiological reports were retrospectively reviewed by two investigators (S.P. and D.P.). The sonographic appearance of the tendon sheaths prior to injection was recorded as containing fluid, fluid with thickening of the tendon sheath, echogenic fluid, increased color-Doppler signal, a combination of characteristics, or no fluid or thickening. The injection approach was recorded as either in or out of plane based on the sonographic images reviewed. All quantitative data was analyzed with descriptive statistics using Excel 2011.

To assess the agreement between the clinical request and injected sites, an injection index (InIx) was developed. InIx was defined as the number of tendons injected divided by the number of tendons requested and was calculated for each procedure.

The procedural records were examined for intra-procedure complications including pain, swelling and bruising. Complications were classified as minor or major according to the Society for Interventional Radiology (SIR) guidelines [12]. The focus of this study was procedural and therefore no long-term follow-up for complications such as subcutaneous atrophy was done.

## Technique

Sonographic guidance is performed on Philips (ATL 5000, iU 22 and Epic) or Siemens (S2000) equipment using linear transducers (14, 15 or 20-MHz). Procedures are performed by staff pediatric interventional radiologists and/or supervised interventional fellows.

Informed consent is obtained and patient pain and anxiety is controlled by either nurse-administered intravenous sedation (fentanyl (Sandoz Inc., Quebec, Canada), midazolam (Sandoz Inc., Quebec, Canada) with possible ketamine (Sandoz Inc., Quebec, Canada)), by an anesthesiologist, or occasionally with no sedation. The sites to be injected are prepared and draped in a sterile fashion. The US transducer is swept along the length of the tendon in both longitudinal and transverse planes to identify a site of peritendinous fluid or signs of inflammation. After identifying a location along the tendon sheath that permits safe injection, a 25-gauge needle is advanced under direct US visualization into the tendon sheath in either the transverse (out of plane) or longitudinal (in plane) approach. In both approaches the needle tip is directly visualized, and it is ensured that it the needle tip is located within the peritendinous space prior to injection. The “out of plane” approach is favored, as it permits to direct and place the needle tip away from the tendon itself, a technique frequently used in interventional radiology when directing needles in limited spaces with vital structures close by (e.g. neonatal brachial vein access). Triamcinolone hexacetonide (20 mg/ml), the preferred steroid (triamcinolone acetonide if former not available, 40 mg/ml), is injected at the dose prescribed by the referring rheumatologist (usually between 5 and 10 mg = 0.25-0.5 ml). This is followed by injection of a similar volume of local anesthetic to clear the needle track and help prevent subcutaneous atrophy (1% lidocaine; Xylocaine, AstraZeneca Canada Inc., Mississauga, Ontario).

## Results

Between May 2006 and April 2016, there were 1275 procedures for joint and/or tendon sheath injections, 350 of which included referrals for tendon sheath injections in 270 patients. In 42/350 procedures the sheaths were not injected due to lack of fluid or inflammatory signs in the tendon sheaths at the time of the procedure. In the remaining 308/350 procedures tendon sheaths were injected in 244 patients. There were 184 females (75.0%) and 60 males (25.0%). Patients ranged in age from 10 months to 17 years and the mean age at time of the procedure was 9 years and 8 months. All patients had a reported diagnosis of JIA.

**Fig. 1:**
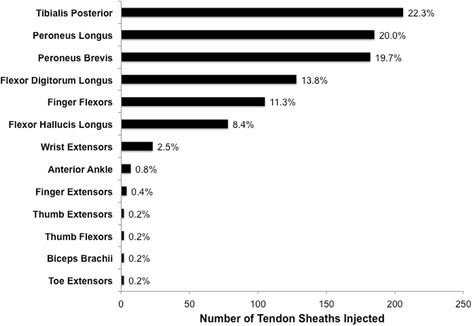
Distribution of specific tendon sheaths injected

A total of 926 tendon sheaths were injected, the distribution of which is presented in Table 1. The most commonly injected anatomical site was the ankle. Specific tendon sheaths are shown in Fig. 1 and average steroid dose injected are presented in Table 2. Procedures involved both tendon sheath and joint injections in 296/308 visits (96.1%), compared to tendon sheaths only in 12 visits (3.9%). On average 3 tendon sheaths were injected per procedure (SD: 2.0, 95% CI: 2.78, 3.22) with 2 tendon sheaths injected most frequently (29.6%). On average 3.4 joints were injected per procedure (SD: 2.5, 95% CI: 3.13, 3.67) with 2 joints injected most often (21.8%).

**Table 1:** Tendon sheaths injected by anatomical site

	Number of tendon sheaths injected	% of Total tendon sheaths
Medial Ankle	412	44.5
Lateral Ankle	367	39.6
Anterior Ankle	7	0.8
Hand	113	12.2
Wrist	23	2.5
Foot	2	0.2
Arm	2	0.2
TOTAL	926

**Table 2:** Average dose of steroid injected

	Average dose, mg (TH)	Average dose, mg (TA)
Medial Ankle		
• Tibialis Posterior (*n* = 206)	7.20	16.43
• Flexor Digitorum Longus (*n* = 128)	6.76	15.00
• Flexor Hallucis Longus (*n* = 78)	6.89	15.00
Lateral Ankle		
• Peroneus Longus (*n* = 185)	7.01	15.00
• Peroneus Brevis (*n* = 182)	6.96	15.00
• Anterior Ankle (*n* = 7)	7.80	12.50
Hand		
• Finger Flexors (*n* = 105)	5.28	10.00
• Finger Extensors (*n* = 4)	7.75	----
• Thumb Extensor (*n* = 2)	8.50	----
• Thumb Flexor (*n* = 2)	7.50	----
Wrist Extensors (*n* = 23)	8.18	20.00
Biceps Brachii (*n* = 2)	20.00	----
Toe Extensors (*n* = 2)	5.00	----

*TH* Triamcinolone hexacetonide (*n* = 298); *TA* Triamcinolone acetonide (*n* = 10)

The majority of patients (78.3%) underwent one procedure during the study period, 17.6% had two, and 4.1% had three or more; 160/244 (51.9%) patients had no prior joint or tendon sheath injections in IR. Of the 53/244 patients who underwent more than one procedure, patients received injections in the same tendon sheaths in 31 instances in a total of 58 tendon sheaths. The ankle region required subsequent injections in the same tendon sheath most often, and an average of 2 tendon sheath injections were required when repeat injections were needed in this region.

**Fig. 2:**
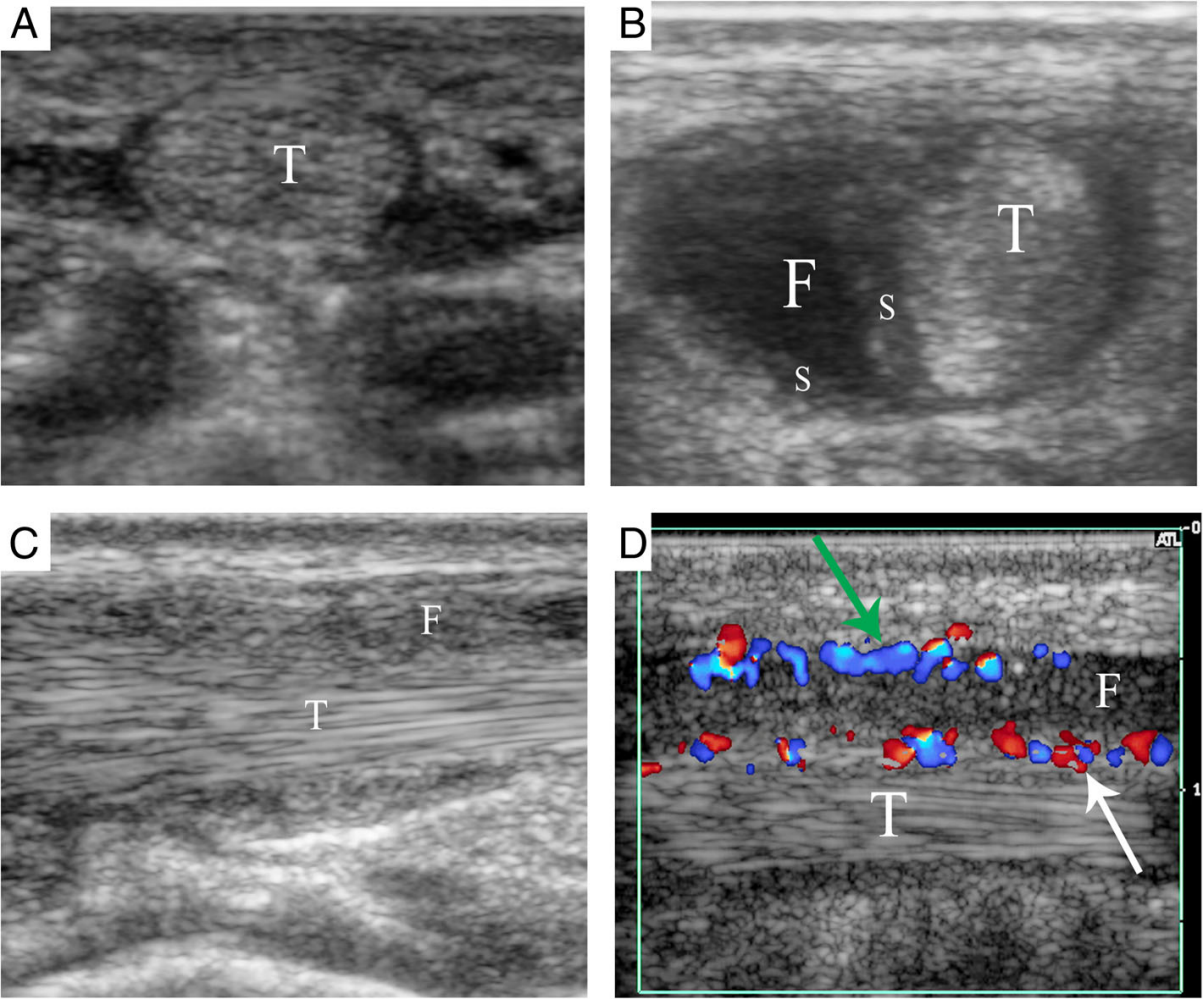
Sonographic findings of tenosynovitis in JIA. **a** 13 years 5 months old female referred for injection of the 4th flexor tendon sheath of the right hand. Ultrasound showed no fluid or signs of inflammation around the tendon in the transverse view. **b** 9 years 11 months old female referred for injection of the tibialis posterior tendon sheath. Ultrasound showed accumulation of peritendinous fluid and thickening of the synovial sheath in the transverse view. **c** 8 years 1 month old female referred for injection of the tibialis posterior tendon sheath. Ultrasound showed accumulation of echogenic peritendinous fluid in the longitudinal view. **d** 15 years 8 months old male referred for injection of the tibialis posterior tendon sheath. Ultrasound showed peritendinous fluid, synovial thickening with increased color-Doppler signal on the synovium covering the tendon (*white arrow*) and lining the cavity (*green arrow*). T = Tendon, S = Synovial sheath thickening, F = Peritendinous fluid

Overall, the mean InIx was 0.765; 148 procedures (48.1%) had an InIx of 1.0, indicating that all tendon sheaths were injected as requested, 98 procedures (31.8%) had an InIx between 0.5 and 1.0, 59 procedures (19.2%) had an InIx < 0.5, and in 3 procedures (1%) the InIx was >1.0. The InIx index was 0 in 42 procedures, indicating that no tendon sheaths were injected, due to a lack of peritendinous fluid or signs of inflammation on US (Fig. 2a).

On US, the tendon sheaths in 283/308 (91.9%) procedures showed peritendinous fluid and synovial sheath thickening (Fig. 2b). Accumulation of peritendinous fluid on its own was seen in 19/308 (6.2%). Echogenic peritendinous fluid was seen in 6/308 (1.9%) (Fig. 2c). Increased color-Doppler signal was seen in combination with peritendinous fluid and synovial sheath thickening in 9 procedures (Fig. 2d), and with echogenic peritendinous fluid in 1 procedure.

**Fig. 3:**
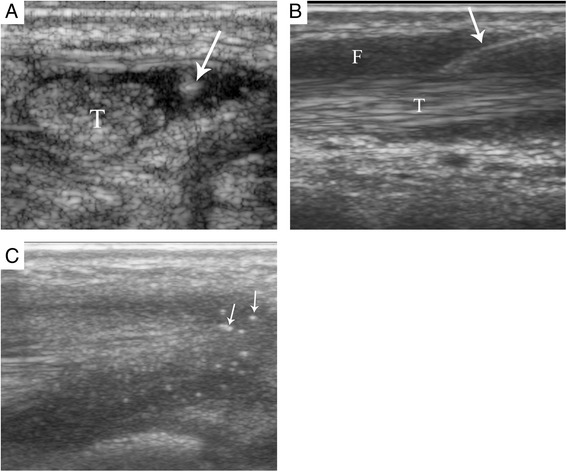
Sonographic images of tendon sheath injection approach. **a** An out of plane injection of the tibialis posterior tendon sheath of a 13 years 8 months old male. **b** An in plane injection of the tibialis posterior tendon sheath of a 9 years 11 months old female. *Arrows* point to the needle. **c** Steroid solution (*small arrows*) surrounding the tibialis posterior tendon of a 9 year 4 months old male after injection. T = Tendon, F = Peritendinous fluid

Approximately two thirds of patients underwent general anesthesia (63.1%), one third had nurse-administered IV sedation (36.0%), and 0.9% received no pain or anxiety management. The average age of patients was 8.6, 11.3 and 16.7 years requiring general anesthesia, IV sedation and no pain management, respectively. Triamcinolone hexacetonide was used in 298 procedures (96.8%), whereas triamcinolone acetonide was used in 10 procedures (3.2%). A 15-MHz linear US probe was used for most procedures (*n* = 265, 86.0%), 14-MHz linear US probe was used in 42 procedures (13.7%) and a 20-MHz linear US probe was used in one procedure (0.3%). An out of plane approach was used in 266 procedures (86.3%) (Fig. 3a), an in plane approach in 32 procedures (10.4%) (Fig. 3b) and a combination of in and out of plane approach was used in 10 procedures (3.3%).

**Fig. 4:**
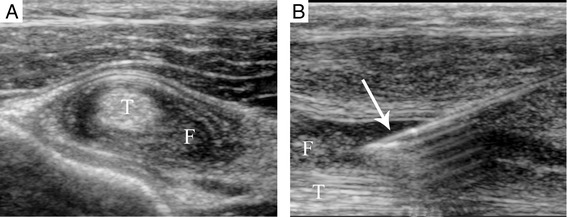
6 years 8 months old male referred for injection of biceps brachii tendon bilaterally. **a** Ultrasound showed echogenic peritendinous fluid and thickening of the sheath around the tendon (T). **b** The fluid was aspirated with a 20-gauge angiocath™ (*arrow*) and sent for culture

Peritendinous fluid was aspirated in 2 procedures. In one case, fluid suspicious of infection by its echogenic appearance was aspirated from the right biceps brachii tendon sheath (Fig. 4), and sent for culture. In the second case, echogenic peritendinous fluid was similarly aspirated from the tibialis posterior tendon sheath followed by injection of steroid.

Two minor intra-procedure complications were reported. Following one procedure bruising around the medial malleolus arose after injection of the medial ankle tendons (SIR A). In another, peritendinous fluid suspicious of infection was seen on US and injection of steroid was deferred (same patient as above). This necessitated another patient visit after subsequent cultures of the fluid were negative. No major complications were reported.

## Discussion

US-guided steroid tendon sheath injections are frequently requested by pediatric rheumatologists to manage children with JIA. The most commonly injected tendon sheaths are the tibialis posterior, peroneus longus and peroneus brevis. In this cohort, the procedure was most commonly performed under general anesthesia, using a 15-MHz linear US probe and 25-gauge needle injecting triamcinolone hexacetonide. The most common sonographic appearance of tenosynovitis in this cohort was accumulation of peritendinous fluid and synovial sheath thickening. There was good agreement between the clinical request and the sonographic signs of inflammation of the tendon sheath.

Tenosynovitis is recognized to occur commonly in the ankle region of children with JIA, with up to 71% of clinically swollen ankles exhibiting sonographic signs of tendon sheath inflammation [5]. Young et al. [13] reported that 75% of the 104 tendon sheath injections were performed in the foot/ankle region in their cohort of patients with JIA. Javadi et al. [6] studied 45 patients with a confirmed diagnosis of JIA and determined using MRI that tenosynovitis was present in 59% of wrists and in 33% of ankles with synovitis respectively, with the tibialis posterior being the most commonly involved. The results of this current study are consistent with previous reports in that tenosynovitis was present in 24% of the referred population and approximately 85% of tendon sheaths injected were in the ankle region, with the tibialis posterior contributing to 22% of all tendon sheaths injected. These current results differ from other studies in that the vast majority of tendon sheath injections were performed in conjunction with intra-articular injections and isolated tendon sheath injections were only performed in 3.9% of procedures, compared to other studies which have reported isolated tenosynovitis in up to 39% of ankles [5]. Of note, isolated tenosynovitis in this patient group was encountered most frequently in the ankle region, with 9/12 procedures involving tendons in this location.

The most frequently employed injection approach was out of plane. This approach provides better visualization of the needle tip within the peritendinous fluid, in the space between the synovial sheath and tendon itself (Fig. 3a). Clear visualization of the needle tip prevents accidental needle entry into the tendon fibers and permits a safe injection.

To the best of our knowledge no large series of US-guided tendon sheath injections in children has been reported, and the sonographic appearance of tenosynovitis in JIA has yet to be described in the literature. Lambot et al. [14] proposed a MRI scoring system for the assessment of tenosynovitis in clinically involved wrists in patients with JIA. In this study, peritendinous fluid accumulation and synovial sheath thickening was observed in the majority of patients with JIA, which is consistent with the OMERACT Ultrasound Task Force’s definition of tenosynovitis in other conditions [8]. Furthermore, a semi-quantitative scoring system in grey-scale and colour Doppler mode US has been developed by the OMERACT US group to assess tenosynovitis [15, 16]. Several studies evaluating this scoring system on patients with RA have shown excellent intra- and inter-reader agreement as well as good ability to detect response to systemic treatment [17, 18]. Moreover, studies evaluating synovitis and tenosynovitis on US in JIA have emerged which outline the usefulness of US in monitoring disease progression, however an US scoring system has yet to be proposed [19, 20].

In this study population there was good agreement between the clinical request and the sites injected. Previous studies have reported a poor inter-observer agreement for clinical examination versus US of the foot in JIA [10]. Pascoli et al. [11] found that clinical examination was inadequate in detecting tenosynovitis in the ankle region, as among 19 lateral ankle tendons thought to be clinically involved, less than 50% had sonographic signs of involvement. As previously stated, a tendon sheath may not be injected safely if sufficient peritendinous fluid is not seen on US. As such, good agreement between the clinical signs of and sonographic signs of tenosynovitis may avoid unnecessary referrals and improve hospital efficiency. In this cohort the average InIx was 0.765, indicating that for every 100 tendon sheath injections referred, 77 are performed. Furthermore, the InIx was equal to 1.0 in 148 procedures, indicating that 48% of the clinical referrals were completed as requested. These results demonstrate that tenosynovitis was adequately detected on clinical exam, and that interventionists can expect to inject exactly as requested approximately half of the time.

This study has several limitations. This was a retrospective single-center study. Occasionally images stored in PACS were suboptimal and images may not have been stored for all tendon sheaths treated. Injections occurring in the Rheumatology clinic were not included in the denominator of 1275 procedures. In this institution tendon sheath injections without ultrasound guidance are occasionally performed in isolated treatment of the flexor compartment of the hand.

The focus of this study was procedural and therefore we did not complete a long-term follow-up of patient outcomes. Additionally, patients’ medical records were not completed with the level of accuracy required to assess the correlation between the intervention and patient outcomes. Furthermore, it was difficult to assess the impact of the intervention compared to a change in the patient’s medical treatment. Most patients required only one procedure and thus we believe this is an indicator of effectiveness in conjunction with the management by the rheumatology team. Additionally, only 31/244 patients (12.7%) required repeat injections into the same tendon sheath and only 58/926 (6.3%) tendon sheaths required a repeat injection. Only 2 minor and no major complications were observed, and thus the procedure was deemed to be safe and exhibit a high technical success rate.

## Conclusions

In conclusion, US-guided tendon sheath injections are a frequent procedure in patients with JIA. It is a safe intervention with a high technical success rate. The ankle region, specifically the medial ankle, is most commonly injected. The most frequent US finding of tenosynovitis in JIA is accumulation of peritendinous fluid and synovial sheath thickening. There is good agreement between the clinical request and the sites injected. These findings might assist rheumatologists, radiologists and other health care professionals involved in the care of patients with JIA, to characterize and more effectively manage tenosynovitis in their patients.

## Abbreviations

InIx: Injection index
IR: Interventional radiology
JIA: Juvenile idiopathic arthritis
RA: Rheumatoid arthritis
US: Ultrasound
